# Measures of Relative Dentary Strength in Rancho La Brea *Smilodon fatalis* over Time

**DOI:** 10.1371/journal.pone.0162270

**Published:** 2016-09-06

**Authors:** Wendy J. Binder, Kassaundra S. Cervantes, Julie A. Meachen

**Affiliations:** 1 Department of Biology, Loyola Marymount University, Los Angeles, California, United States of America; 2 Department of Anatomy, Des Moines University, Des Moines, Iowa, United States of America; Monash University, AUSTRALIA

## Abstract

The late Pleistocene megafaunal extinction of approximately 12,000 years ago, included the demise of *Smilodon fatalis*, a hypercarnivore from the Rancho La Brea deposits, which has been studied across time by looking at different deposits or pits to determine morphological size and shape changes and trends during this time. To better understand functional aspects of these changes, this study focused on a measure of jaw strength over time, which can give an indication of morphological changes within the jaw that cannot be seen using surface morphometrics. By radiographing dentaries, cortical bone can be seen, which provides an estimate of resistance to bending forces while biting, and can be measured and used as an indicator of jaw strength. Measurements were taken at repeatable locations on the dentary of the depth of the cortical bone, and of a standardized measure of cortical bone, which allows for the comparison between different individuals. Specimens included those of five different pits ranging from about 37 Kybp to 13 Kybp (just before the extinction of *S*. *fatalis*). No significant difference was found in the depth of jaws at any of the measurement points from any of the pits. However, significant differences were found in both the actual thickness of cortical bone, and the standardized thickness of cortical bone at the lower P4 between pit 13 (which had the lowest amount of bone) and pit 61/67 (which had the highest). These conclusions support other studies that have shown that individuals in pit 13 were under physiological and perhaps dietary stress, which may be reflected in the deposition of cortical bone, while the opposite trend is seen in the individuals in pit 61/67. Our results further support findings suggesting *Smilodon* did not appear to be morphologically most vulnerable right before its extinction.

## Introduction

The well-preserved natural laboratory of Rancho La Brea (RLB), spanning 30,000 years over the late Pleistocene and into the Holocene, is an excellent site to examine ecological and environmental changes, and how these changes led into the end-Pleistocene extinction event and the Pleistocene-Holocene transition. This site is also well-known for its disproportionate numbers of big carnivores, with large numbers of dire wolves, *Canis dirus*, and sabertooth cats, *Smilodon fatalis*. These large sample sizes give us the opportunity to examine morphological changes in carnivore species over the course of the late Pleistocene approaching the extinction event. *Smilodon fatalis* shows well-documented morphological changes in crania over its late Pleistocene tenure at Rancho La Brea [1, 2]. These changes are likely linked to both climate and other environmental causes [1], and give us ecological insight into *S*. *fatalis’* prey-killing behavior and interactions with other species over this time interval.

A few pits at RLB are of particular importance for ecological understanding because of their co-occurrence with major climatic or extinction events. Pit 13 is of special interest at RLB as carnivores from this pit show increased tooth breakage and wear that occurred during their lifetime [3, 4]. The six known radiocarbon dates from this pit, between 17,000–18,500 years before present [5, 6], suggest this pit was deposited during the last glacial maximum (LGM) in western North America. [7]. The intense breakage and wear from pit 13 implies that carnivores at RLB were undergoing ecological and physiological stress during this period of cooling in Earth’s history [3, 4].

Pit 61–67 is another important deposit at RLB. This pit contains the largest number of carnivore specimens and is the latest known Pleistocene deposit at the site. The seven known radiocarbon dates place this deposit between 5,000–14,300 years before present with five of those dates falling between 13–14.3 Kybp, thousand calibrated years before present [5]. These older dates place the deposition of pit 61–67 concurrent with the end-Pleistocene extinction events [8]. Surprisingly, *S*. *fatalis* specimens from pit 61–67 are robust, large, and show no indication of ecological or physiological stress [1, 4, 9].

*Smilodon fatalis* mandibles change over time throughout the late Pleistocene at RLB with fluctuations between less and more derived sabertooth-like morphologies. In Meachen et al. [1], *S*. *fatalis* mandibles from pit 77 (32–40 Kybp) and from pit 2051 (approx 23–25 Kybp) show ancestral sabertooth-type morphology with small gracile mandibles that have higher coronoid processes and smaller mandibular flanges; whereas mandibles from pit 91 (approx. 27–28 Kybp) [6] and pit 61–67 (13–14 Kybp) show derived sabertooth morphologies with larger size, more robust mandibular corpora, shorter coronoid process and larger mandibular flanges. Pit 13 (17–18.5 Kybp) showed intermediate morphologies between the two extremes [1]. Although these changes are visible in the external osteological measurements, we may get even more information from the radiographic cross-sections.

Radiographs can show the cortical structure of the mandible, which can be modeled as a hollow beam. This can give a good indication of bending strength and resistance to torsion during killing and feeding [10–12]. Radiographic cross-sections can give us additional information that cannot be gleaned from external measurements such as how the forces applied to the mandible have evolved in *S*. *fatalis*. Mandibular resistance to bending is governed by killing and feeding behaviors in carnivores and can evolve over time, such as a shift in a preferred prey species, a change in prey-killing behavior involving the crania, or different tissues (e.g., flesh versus bone) that *S*. *fatalis* might ingest.

Using microwear DeSantis, Schubert (9) determined that *S*. *fatalis* consumed bone at Rancho La Brea, but the proportion of bone consumption may have changed over time as environmental conditions changed. Consumption of hard foods, such as bone, can be reflected in mandibular cortical bone thickness in carnivorans, as the mandibular corpus continues to remodel throughout life [11, 13]. We would not expect a sabertooth cat to show the same cortical thickness as a durophagous specialist, like a hyena; however, when comparing the cortical thickness within a species over time, we do expect to find nuanced differences in cortical thickness relative to dietary changes.

Here, we examine whether the results from our previous studies on *Smilodon fatalis* pertaining to mandibular shape change through time [1] and tooth breakage and wear [4] are reflected in the cortical structure of the mandible. If the external morphology is echoed in the cortical bone we might expect to see increased dorsoventral thickness in mandibles from pits 91 and 61–67, thinner cortical bone in mandibles from pit 77 (we did not measure pit 2051 for logistical reasons) and intermediate values in pit 13, which have intermediate mandibular morphology. Alternatively, results from tooth breakage and wear studies suggest that we will find the highest amount of cortical thickness in individuals from pit 13, as teeth from that pit show the greatest amount of wear [4].

## Materials and Methods

Data was collected from *S*. *fatalis* hemi-mandibles from the La Brea Tar Pits and Museum collection, from pits 61–67, 13, 3, 91 and 77 (see Table 1). These pits were chosen to match the previous study by Meachen et al. [1], and differences in sample size are a product of the number of available dentaries. Pit 13 had the lowest overall sample size and only 2 dentaries from pit 13 contained canine teeth. A portable x-ray machine was used to take lateral radiographs of individual dentaries, and radiographs were taken using radiographic film in rare-earth cassettes, which magnify and clarify the images. Dentaries were positioned buccal side downward on the cassettes for consistency. While an occlusal radiograph of the dentaries could also have been taken to allow for estimates of bone thickness on the medial and lateral sides, we chose not to take an x-ray in that plane as *Smilodon* hemi-mandibles are heavy (impregnated with asphalt) bent bones that are unstable on their ventral margins, which would introduce a large amount of variance (and error) due to positioning on a very small measure. As the vast majority of the bone occurs in the ventral portion visible from the lateral view, we chose to use the two measurements in that plane alone. All measurements were taken from radiographs placed upon a light table using digital calipers to within 0.01mm.

**Table 1:** Pit age ranges and maximum sample sizes of *S*. *fatalis* dentaries per pit.

Pit	Age Range (Kypb)	Overall sample size
61/67	13–14	24
13	17–18.5	8
3	14–24	27
91	27–28	19
77	32–40	24

**Fig 1:**
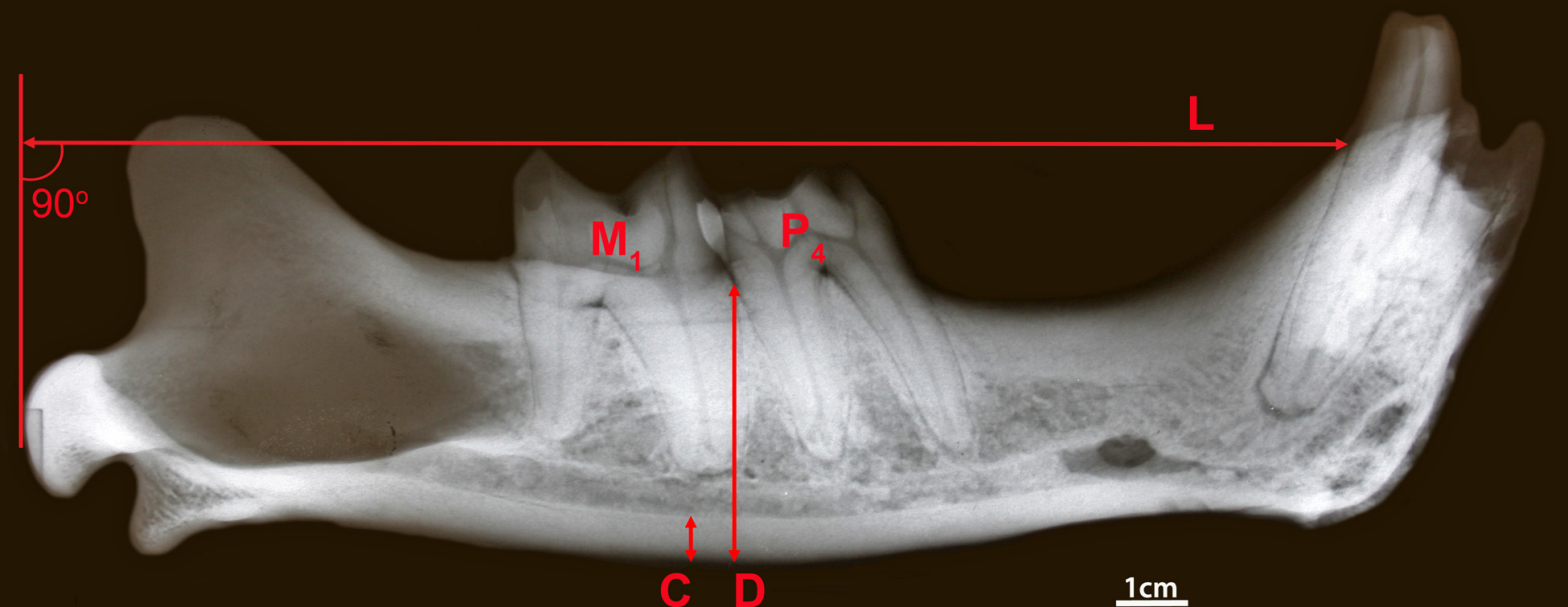
Radiograph of mandible of *S*. *fatalis*, with the relative cortical width, Cort, measurement (C, the cortical bone width); D, the mandible width, and L, the jaw length (posterior condyloid to the posterior alveolar emergence of the canine).

Measurements of dentary dorsoventral thickness and cortical bone dorsoventral thickness were recorded in relationship to the borders of the cheek teeth, including posterior to M1, P4 and anterior to P4, which is denoted P3 (although P3 wasn’t present in our sample), which serve as repeatable landmarks [10]. Cortical and cancellous bone can be visibly differentiated, due to their different densities in radiographs, and the denser cortical bone which is concentrated on the ventral side of the dentary was measured from the ventral border of the dentary to the top of the cortical bone mass at each junction, 90 degrees to the long axis of the dentary; all axes were estimated prior to measurements to consistently measure the depth angle (Fig 1). Dentary height measurements (from the ventral border to the alveoli, including both the cortical and cancellous bone) were also taken in the same locations, 90 degrees to the long axis of the jaw (Fig 1). The mandibular height measurements were used to give a standardized estimate of cortical width (measured as cortical/mandibular thickness).

This measure was based upon and modified from a hollow symmetrical model of the mandible using a linear measurement, a technique previously determined to give a consistent indication of cortical bone thickness [10] and estimate strength in bending rigidity. Although this model is less accurate than a hollow asymmetrical model [10], the difference between the two models is limited (the hollow symmetrical model has been shown to deviate from true second moments of area by 7–16%, while the hollow asymmetrical model deviates by 4–8%), and using a symmetrical model was the most feasible in this situation given that we could not destroy nor CT scan the dentaries, and estimating distances in the mesio-distal plane would have introduced a variable and unreliable measurement. In this study we aim to estimate relative dentary strength through cortical bone thicknesses among *S*. *fatalis* individuals in different pits, rather than to attempt to measure absolute bending strength in the dentary, and therefore we are looking for nuanced differences in cortical thickness through time.

Dentary length measurements were taken from the posterior tip of the condyloid to two different locations: the anterior tip of the alveolar point of emergence of the canine, and the posterior alveolar point of the emergence of the canine. The former measure includes the canine, while the later measure doesn’t; however as canines were very often missing from the dentaries, and due in part to the anterior curvature of the bone, the posterior canine border is often the most anterior distance that can be consistently measured from the radiographs due to the shape and orientation of the mandible. Statistical analyses including ANOVA and *post-hoc* Tukey’s tests were done with SPSS v22.

## Results

**Fig 2:**
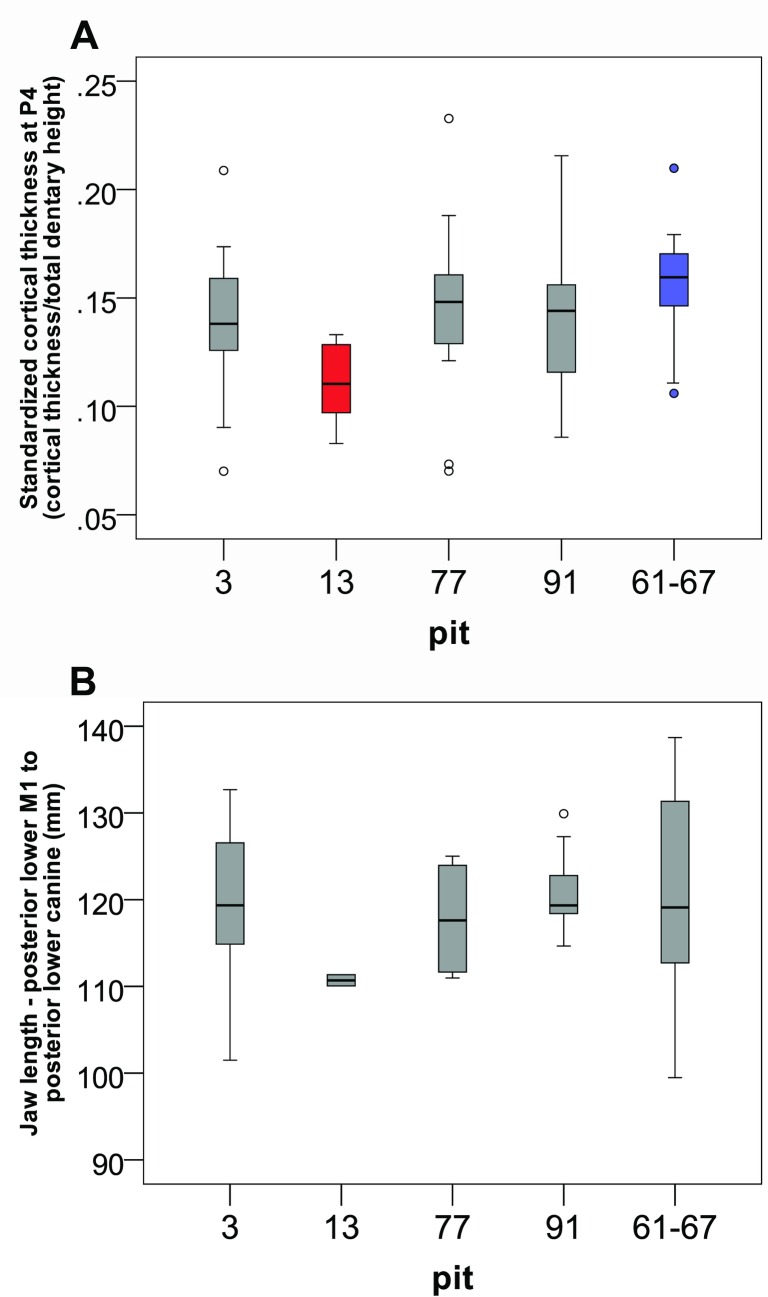
Distribution of measures by pit. Fig 2A shows the different distributions of the values of standardized cortical thickness at P4 in each pit (pit 3, with the lowest value is in red, while pit 61/67 in blue has the highest), and Fig 2B shows the different distributions of jaw length in each pit.

Sample sizes per pit and mean measurements of jaw length and standardized cortical thickness are shown in Table 1. Our cortical ANOVA results do not show significant differences in cortical thickness nor percent cortical thickness (cortical/total dentary length) at posterior M1 or P3, but our ANOVA results do show a significant difference at P4. This difference at P4 is only seen between pits 13 and 61–67. The dentaries from pit 61–67 are significantly thicker than those from pit 13, but all of the other pits fall in between these two extremes (Table 2, Fig 2A). Interestingly, the trend in the mandible lengths is exactly the opposite of the cortical thicknesses. For jaw length including the canine, pit 61–67 still has the longest values, but it is followed closely by pit 13 with the second longest values (Fig 2B). All of the other pits show significantly shorter jaw lengths. The values for the jaw lengths excluding to the canine, however show different results more in line with the cortical measurements (Table 2). Sample sizes are low for some pits which may be problematic.

**Table 2:** Measurements of *S*. *fatalis* dentaries showing significant differences between individual pits (from Tukey’s *post-hoc* analyses of ANOVAs).

Measurement	Pit	Sample size	Mean	Significant differences (and p-values)*
Lower jaw length (excluding canine) (mm)	3	23	**105.19**	**61–67 (0.003)**
	13	7	109.03	none
	77	17	**101.52**	**61–67 (<0.001)**
	91	19	**105.77**	**61–67 (0.014)**
	61–67	24	**113.68**	**3, 77, 91 (see above)**
Lower jaw length (including canine) (mm)	3	20	**119.91**	**61–67 (0.002)**
	13	2†	128.45	none
	77	4	**117.8**	**61–67 (0.050)**
	91	10	**120.81**	**61–67 (0.049)**
	61–67	22	**125.97**	**2, 77, 91 (see above)**
Cortical thickness–P4	3	27	5.52	none
	13	7	**4.64**	**61–67 (0.041)**
	77	20	5.96	none
	91	18	5.86	none
	61–67	23	**6.25**	**13 (see above)**
Standardized cortical thickness–P4	3	27	0.1403	none
	13	6	**0.1103**	**61–67 (0.009)**
	77	19	0.1459	none
	91	18	0.1412	none
	61–67	23	**0.1557**	**13 (see above)**

*Only p-values less than 0.10 are reported here. For significant differences means and p-values are in bold.

† small sample size here for pit 13, which likely contributes to the lack of significant difference between this pit and others.

## Discussion

Our results show interesting findings for the sabertooth cat populations at Rancho La Brea. Although this study, along with another [1] have shown there is microevolution present spanning all of the time-periods that are captured at this site, pits 13 and 61–67 show the most interesting patterns. *S*. *fatalis* from pit 61–67 is consistently the largest and most robust, including its cortical thickness [1, 2]. This suggests that *S*. *fatalis* leading up to the extinction event were healthy and thriving. Large mandibles suggest that individuals are increasing in size, and robust, cortically thick dentaries support feeding on large prey that produce forces from which there would need to be sufficient resistance, increasing bone deposition in the dentary. These assertions from pit 61–67 have been backed up by several studies of Rancho La Brean megafauna, showing that the pit that was deposited directly before the extinction event was not experiencing environmental stresses [1, 4, 9, 14]. This time in Southern California was one of warming and drying [15], which would suggest that *S*. *fatalis* became larger and more specialized during warm, dry times [1].

*Smilodon fatalis* from pit 13 show a different result. Sabertooth cats from this pit demonstrate evidence of environmental stress. Although they have longer mandibles than *S*. *fatalis* from pits 3, 77, and 91, they have the lowest relative cortical thickness. This might suggest that they are processing softer foods than their conspecifics in different pits [11], which would contradict the findings of Binder and Van Valkenburgh [4] who found that *S*. *fatalis* in pit 13 had extreme tooth wear and breakage, suggesting a hard diet incorporating more bones.

One explanation for this seeming contradiction could be nutritional stress. A study on rats showed that offspring who were gestated by and suckled from a protein deficient mother showed decreased cortical axis moment of inertia in the mandible even into early adulthood when compared to well-nourished offspring [16]. Other studies examining bone densities in offspring of malnourished mothers have found a similar result [17, 18]. A 2010 study also showed that perinatal nutritional conditions are so important to bone density and health that if given nutritional supplements later in life, individuals cannot fully recover from these effects even as adults [19]. In contrast, a study that showed that nutritional stress applied to adult individuals (who were healthy neonates) produces no difference in bone density or cortical area [20]. Further, protein malnutrition across generations has been shown to cause variations in size and other cranial parameters in rats, affecting multigenerational growth trajectories [21]. These results, taken together, suggest that pit 13 *S*. *fatalis* may have not only been experiencing increased competition during their lifetimes (evidenced by greater tooth breakage and wear), but they may have inherited a generational nutritional deficit from their mothers in utero and during their early development, from which they couldn’t fully recover even as adults, resulting in thinner, but similarly distributed cortical bone.

The timing of the deposition of pit 13 may hold some clues to this nutritional deficit. The six known dates for pit 13 are 18.412, 18.569, 18.111, 17.149, and 17.394 thousand years before present and one uncalibrated outlier of 7.665 thousand radiocarbon years old. These five clustered dates between 17–18.5 thousand years ago coincide with the last glacial maximum in western North America [7]. This time of relative cold could have put stresses on mammal populations. A study showed that during times of cold stress herbivore populations are at lower densities, which puts stress on carnivore populations [22]. However, this study looked at populations in a very cold locality (Norway), and even in times of relative cold the Los Angeles basin may not have experienced the same types of effects. A high amount of *Pinus* pollen in lake sediment cores from Southern California during the last glacial maximum indicate a cool and wet environment, with higher precipitation variability, but overall increased precipitation relative to the early Holocene [15]. Not many studies have examined the complex interplay between precipitation and carnivore competition. However a few studies have examined the effects of precipitation on mesopredators (opossums in one case and smaller carnivorans in another) and found that variation in precipitation has a rather large effect–greater than mean annual temperature–on the hunting activities of these smaller carnivores [23, 24]. Vegetational changes that accompany changes in temperature and precipitation at this time in Southern California (*i*.*e*., invasion of pine forest), most certainly would affect the types of prey that would be present, which would have in turn affected *Smilodon* populations at Rancho La Brea. One final possibility to consider is that our small sample size of individuals from pit 13 (n = 7) may be anomalous and not truly indicative of the entire *Smilodon* population from that time period. However, our results indicate that most of our specimens show the same trends and a pattern of small cortical thickness relative to length for a population of animals that show signs of extensive carcass processing behavior [4].

## Conclusions

This study focused on a measure of jaw strength over time by radiographing *Smilodon* dentaries. This provides an estimate of cortical thickness and can be used as an indicator of jaw strength. Using specimens from five different pits ranging from about 37 Kybp to 13 Kybp (just before the extinction of *S*. *fatalis*), significant differences were found in both the actual thickness of cortical bone, and the standardized thickness of cortical bone at the lower P4 between pit 13, which had the lowest amount of bone, and pit 61/67 which had the highest. This supports other studies that have shown that individuals in pit 13 were under physiological and perhaps dietary stress, which may be reflected in their deposition of cortical bone, while the opposite trend is seen in the individuals in pit 61/67. This opens the door for future studies on the effects of nutritional stress in *S*. *fatalis* and other carnivores at Rancho La Brea and throughout the Pleistocene. The unique sample of juvenile *S*. *fatalis* present at Rancho La Brea would be excellent for a continuation of this work.

## Supporting Information

S1 Dataset *Smilodon fatalis* measurement data by pit. (XLSX)
